# Relationship Between COVID-19 and Retinal Vein Occlusions

**DOI:** 10.1155/joph/6507997

**Published:** 2025-09-29

**Authors:** Tetsuya Muto, Shigeki Machida, Shinichiro Imaizumi, Koju Kamoi

**Affiliations:** ^1^Department of Ophthalmology, Dokkyo Medical University Saitama Medical Center, Koshigaya, Japan; ^2^Department of Ophthalmology, Imaizumi Eye Hospital, Koriyama, Japan; ^3^Department of Ophthalmology, Fukushima Medical University, Fukushima, Japan; ^4^Department of Ophthalmology, and Visual Science, Institute of Science Tokyo, Tokyo, Japan

**Keywords:** branch retinal vein occlusion, central retinal vein occlusion, coronavirus disease 2019, severe acute respiratory syndrome coronavirus 2

## Abstract

The relationship between coronavirus disease 2019 (COVID-19) infection or vaccination and retinal vein occlusions (RVOs) remains controversial. RVOs include central and branch RVOs. Previous studies have indicated a link between RVOs and COVID-19. RVOs develop when the retinal blood vessels are clogged by thrombin or lipid deposition. The retina, an important component of the visual apparatus, relays the visual information to the brain after light stimulation. When retinal veins are clogged, the damage can range from slightly reduced vision to complete blindness. SARS-CoV-2, the causative agent for COVID-19, leads to endothelial dysfunction and increased von Willebrand factor (VWF) antigen levels in the blood, which activate the coagulation process and platelet aggregation. Activation of tissue factors initiates the coagulation cascade, leading to fibrin formation through thrombin. Because arteries and veins sometimes cross in the retina, the vein, with its thin vessel wall, may be compressed. As a result, blood flow slows due to venous constriction, and clotting is more likely to occur at the crossing point. RVO ultimately develops through these processes. Patients with COVID-19 have significantly elevated levels of VWF antigen and activity, which likely contribute to the increased risk of thrombosis observed in COVID-19-associated coagulopathy. As RVOs align with conventional approaches, ophthalmologists should consider COVID-19 as a potential etiological factor when evaluating patients presenting with acute vision loss. Enhanced awareness of this association may facilitate timely diagnosis and tailored patient care in affected populations.

## 1. Introduction

Retinal vein occlusions (RVOs) are retinal vascular disorders characterized by dilatation of retinal veins, retinal and subretinal hemorrhages, macular edema (ME), and varying degrees of retinal ischemia [1]. RVOs are classified into branch RVO (BRVO), hemi-central RVO (hemi-CRVO), and central RVO (CRVO), depending on the site of obstruction [2]. When the occlusion occurs within or posterior to the optic nerve head, it is classified as CRVO; when it occurs at a major bifurcation, it is considered hemi-CRVO; and when it involves a tributary vein, it is BRVO. Hemi-CRVO is often regarded as an intermediate condition between BRVO and CRVO [3]. Together, RVOs represent the second most common cause of retinal vascular blindness after diabetic retinopathy [2]. BRVO is more frequent than CRVO, with estimated worldwide prevalences of 0.4% and 0.08%, respectively. Both occur equally in men and women, with risk increasing with age [4]. The development of RVO is closely associated with systemic risk factors, particularly hypertension and hyperlipidemia; diabetes mellitus is a less common contributor [5]. Currently, no treatment reliably restores perfusion. Although serial intravitreal antivascular endothelial growth factor (VEGF) therapy is the standard of care for ME, grid laser photocoagulation and intravitreal corticosteroids remain reasonable options in selected cases [1, 2]. These features of RVOs were well established prior to the coronavirus disease 2019 (COVID-19) pandemic.

COVID-19, which is caused by severe acute respiratory syndrome coronavirus 2 (SARS-CoV-2), has major health implications and continues to burden healthcare systems worldwide [6]. Some patients with COVID-19 infection also experience multiorgan pathology and vascular damage [7], increasing the risk of various fatal vascular occlusive diseases, such as thrombosis, myocardial infarction, arrhythmia, and cerebral apoplexy [8–11]. Increasing thrombotic tendencies and increased risk of thrombosis persist for several months after COVID-19 infection [12].

To prevent the further spread of COVID-19, vaccine development was accelerated at an unprecedented pace. However, side effects were reported in 50%–90% of participants in randomized clinical trials of COVID-19 vaccines [13]. The concept of vaccine-induced immune thrombotic thrombocytopenia is crucial for addressing vaccine skepticism [14]. Adverse events related to COVID-19 vaccination are generally categorized as local side effects, systemic side effects, or other reactions such as allergies [15]. Thus, vaccination may also carry a risk of local ocular thrombosis.

The retina and its vasculature could be directly accessed with fundus photography, which is a rapid and noninvasive method, and fundoscopic examination of the ocular fundus. The retinal arteries and veins may represent the state of the body's entire microvascular system [16]. Therefore, ophthalmologists should check for any vascular abnormalities following COVID-19 infection or vaccination. However, whether the increased risk of thrombosis with the cause by COVID-19 or vaccination is associated with the development of RVO remains unclear.

RVOs involve CRVO and BRVO. RVO is the most common retinal vascular disease after diabetic retinopathy [17]. Currently, the global prevalence of RVOs and CRVOs is 13/1000 and 5/1000, respectively [18].

Sudden visual impairment is common in CRVO, whereas visual field defects and shape distortions are typical symptoms in BRVO. RVOs affect visual function and are characterized by retinal hemorrhage, soft exudates, and macula edema. CRVO can be classified as ischemic or nonischemic. Ischemic CRVO can cause neovascular glaucoma and blindness. Anti-VEGF antibodies and retinal photocoagulation are the primary treatment methods for CRVO [19–21]. Since anti-VEGF antibodies were introduced, the prognoses of these conditions significantly improved [21].

RVOs are relatively common disorders that can cause mild to severe visual impairment. The risk factors for RVOs include hypercoagulability and thrombotic disorders. In this review, we examined reports published between 2020 and 2023 that investigated the relationship between COVID-19 and RVOs.

## 2. Mechanism of Vascular Occlusion of COVID-19

**Figure 1:**
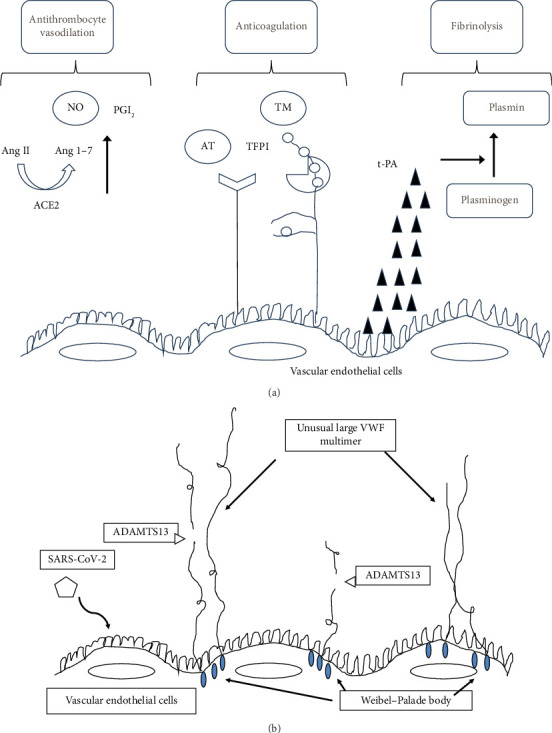
(a) Images of the antithrombotic system. The vascular endothelium has three antithrombotic systems for the most part. Antiplatelet action and vasodilator action: angiotensin II is resolved by angiotensin-converting enzyme 2 and produces angiotensin-converting enzymes 1–7. Angiotensin-converting enzymes 1–7 activate nitrogen monoxide and prostaglandin *I*_2_. Anticoagulant action: glycocalyx sprouts on the vascular endothelium. The anticoagulant action increases after antithrombin, a plasma protein and tissue factor pathway inhibitor, is combined with heparan sulfate on the vascular endothelial cell surface. Thrombomodulin shows an anticoagulant action through the activation of protein C. Fibrinolytic action: endothelial cells activate fibrinolysis by releasing the tissue plasminogen activator. (b) Once vascular endothelial cells are infected by SARS-CoV-2, their Weibel–Palade bodies (WPBs) release von Willebrand factor (VWF). VWF assembles into unusually large multimers composed of > 200 molecules. Under normal conditions, these multimers are rapidly cleaved by ADAMTS13. However, due to the disrupted balance between VWF and ADAMTS13 in SARS-CoV-2 infection, the excessively large VWF multimers are not efficiently cleaved to the appropriate size, thereby facilitating thrombin formation.

Figure 1 shows the mechanism of vascular occlusion in COVID-19. SARS-CoV-2, the causative agent for COVID-19, invades cells by attaching to angiotensin-converting enzyme 2 (ACE2) [22, 23]. COVID-19-associated coagulopathy increases the generation of thrombin, a coagulation factor, in the veins and arteries, which is strongly correlated with vascular endothelial damage [24, 25]. Additionally, vascular endothelial cells infected with SARS-CoV-2 tend to release von Willebrand factor (VWF) and angiopoietin 2. The VWF causes platelets to adhere to the connective tissue present under the layer of vascular endothelial cells, providing a foothold for thrombin [26]. In severe COVID-19, dysregulation of the VWF–ADAMTS13 axis plays a pivotal role [27]. Endothelial Weibel–Palade bodies release unusually large VWF multimers more rapidly than they can be cleaved by ADAMTS13, the metalloprotease that regulates VWF multimer size [27]. Patients with COVID-19 exhibit markedly elevated VWF antigen levels and activity, accompanied by reduced ADAMTS13 activity, resulting in an excessive VWF/ADAMTS13 ratio [27, 28]. This imbalance promotes a hypercoagulable state with the formation of platelet-rich microthrombi. The phenomenon is analogous to thrombotic microangiopathy: under normal conditions, ultralarge VWF (ULVWF) strings released from the endothelium are rapidly cleaved by ADAMTS13 to prevent uncontrolled platelet adhesion. In COVID-19, however, massive VWF release combined with reduced ADAMTS13 activity leads to persistence of ULVWF multimers, driving widespread thrombosis [29]. Notably, baseline plasma VWF levels are not typically elevated in RVO patients without COVID-19 [29], suggesting that the extraordinary VWF elevation and endothelial injury in COVID-19 may contribute to RVO development in this setting. Angiopoietin 2 increases inflammation and exacerbates apoptosis and the permeability of vascular endothelial cells [26]. Moreover, infection in these cells decreases antithrombogenicity. Inflammatory macrophages secrete inflammatory cytokines as defense against viral infection. Subsequently, crosstalk between inflammatory and coagulation factors further reinforces inflammation and coagulation. SARS-CoV-2 can also directly infect macrophages, which leads to the expression of tissue factor, activating the exogenous blood coagulation system. Crosstalk between inflammatory and coagulation factors causes platelet and neutrophil activation. Subsequently, neutrophil extracellular traps are released from the activated neutrophils, leading to a coagulation cascade, reducing ACE2 levels on the cell surfaces, creating an ideal microenvironment for thrombin production.

### 2.1. CRVO After COVID-19 Infection

Table 1 and S1 show the published cases of CRVO development following a COVID-19 infection [30–45].  Table S1 is included in the Supporting Information. The mean age was 36.4 ± 11.0 years (range: 15–54), which was significantly lower than the mean age of 71.2 ± 12.0 (range: 21–92) years from our unpublished data (*p* < 0.001, unpaired *t*-test) collected before the COVID-19 pandemic. This difference indicates that COVID-19 infection can cause CRVO at an earlier age than expected. The total number of cases reported in the included studies was 27, comprising 16 men and 11 women. The incidence of CRVO was not significantly different between the two sexes (*p*=0.38, unpaired *t*-test). In comparison, previous data included 517 CRVO cases, with 296 men and 221 women [46]. Li et al. reported that CRVO occurs more frequently in men (50.4%) than in women (49.6%) before the COVID-19 pandemic [47]. Laterality revealed that eight, five, and two patients developed CRVO in the right, left, and both eyes, respectively. Li reported a left-eye onset preference in CRVO development (right: 30.0%, left: 31.2%, both eyes: 6.3%, unspecified: 32.5%) [47]. The time between the COVID-19 diagnosis and CRVO symptom onset was 0–6 months, making it difficult to determine the point at which CRVO is a complication of SARS-CoV-2 infection. There was no fixed period when the blood test results returned to normal following SARS-CoV-2 infection. Grover et al. reported that patients with the best-corrected VA (BCVA) of counting fingers were assigned a logarithmic minimum angle of resolution (logMAR)value of 2.6; hand motion, 2.7; light perception, 2.8; and no light perception, 2.9 [48], and we followed this arrangement. The mean logMAR BCVA at the initial visit was 0.73 ± 0.75 (range: 0–2), which was not significantly different from that found in previous data (0.72 ± 0.55; range: −0.18-2.30) [46]. Intravitreal injection of anti-VEGF antibodies is the most common treatment for CRVO; however, systemic corticosteroids have already been administered to patients whose CRVO symptoms appeared within a few days of COVID-19 infection. Although photocoagulation is occasionally performed to treat CRVO, none of the abovementioned cases underwent this procedure. Sen et al. reported that younger age affected visual outcomes in CRVO treated with intravitreal injection of anti-VEGF antibodies [49]. Furthermore, in a sample comprising 85 patients with CRVOs and 26 with BRVOs, Dărăbuş et al. revealed that age and baseline BCVA were the most important nonimaging predictors of BCVA after RVOs [50]. Thus, the lower mean age in this study may have affected the visual outcomes.

### 2.2. CRVO After COVID-19 Vaccination

Tables 2, 3, and S2 show published cases of CRVO that developed after COVID-19 vaccination [51–62].  Table S2 is provided in the Supporting Information. The mean age in this subset of patients was 39.4 ± 13.3 years (range: 13–54), which was not significantly different from that of patients who developed CRVO after a COVID-19 infection (Tables S1 and 2) (*p*=0.45, unpaired *t*-test). Thirteen cases developed postvaccine CRVO, comprising nine men and four women. The sex ratio of patients with postvaccine CRVO did not differ significantly from that of patients with postinfection CRVO (*p*=0.79, unpaired *t*-test). The time between COVID-19 vaccination and CRVO symptom onset ranged from 0 to 25 days, with a mean of 9.1 ± 7.3 days (range: 0–25).

Table 2 shows the vaccine types administered to each patient. However, owing to the relatively small number of reported cases, determining whether the risk of CRVO differs between different vaccines is difficult. The number of COVID-19 vaccine doses that patients had received before the CRVO onset was 1–3 (mean, 1.8 ± 0.7).

Blood collection was not fixed. Based on a previous report, the logarithmic VA at the initial visit was 0.81 ± 0.73 (range: 0–2) [48], and no significant difference was observed (Table 3) from CRVO after a COVID-19 infection (Table 1) (*p*=0.78, unpaired *t*-test).

Although the main treatment was intravitreal injection of anti-VEGF antibody, some cases had whole-body administration of corticosteroids (Table S2). Only one case was treated with retinal photocoagulation. The mean final logarithmic VA was 0.18 ± 0.31 (range: 0–1), with no significant difference compared with that of patients with post-COVID-19 infection CRVO (Table 1) (*p*=0.78, unpaired *t*-test).

### 2.3. BRVO After COVID-19 Infection

**Figure 2:**
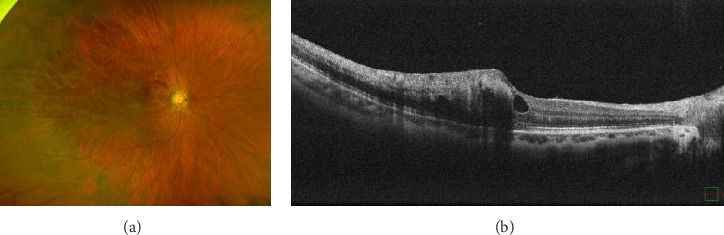
Images of a 47-year-old female who developed branch retinal vein occlusion 3 weeks after COVID-19 diagnosis. (a) Retinal hemorrhage caused by failure of the supratemporal vein in the patient's right eye; (b) retinal edema and an intraretinal cyst that developed alongside the retinal hemorrhage. The optical coherence tomography image is shown in the horizontal plane.

Figures 2(a) and 2(b) show the representative images of the patient with ME secondary to BRVO after COVID-19 infection. This patient was injected with intravitreal aflibercept to improve ME.

Table 4 and S3 show the subset of patients who developed BRVO after a COVID-19 infection [62–68]. Table S3 is provided in the Supporting Information. Their mean age was 56.8 ± 11.7 years (range: 36–74), which was extremely close to 58.2 years in a sample of 354 patients with BRVO reported by Lee et al. in a study conducted before the COVID-19 pandemic [69]. Li et al. and Lee et al. reported a slightly higher incidence of BRVO among women (54.5% and 58.8%, respectively) [47, 69]. The male:female ratio was 5:3. The ocular laterality in those with BRVO after a COVID-19 infection was one right eye and six left eyes. Li et al. reported a right-eye onset preference in the development of BRVO (51.0%) [47]. The mean time between COVID-19 diagnosis and the initial BRVO symptom onset was 50.6 ± 41.2 days (range: 7–90). No patterns were observed when blood test abnormalities returned to normal levels.

The logarithmic VA at the initial visit was 0.67 ± 0.66 (range: 0–2), referring to the previous arrangement [48]. For patients with a post-COVID-19 infection BRVO, the main treatment was intravitreal injection of anti-VEGF antibodies. The final logarithmic VA of this subset was 0.38 ± 0.42 (range: 0–1) (Table 4).

### 2.4. BRVO After COVID-19 Vaccination

Tables 5, 6, and S4 show cases that developed post-COVID-19 vaccination BRVO [70–79].  Table S4 is provided in the Supporting Information. Their mean age was 55.5 ± 13.2 years (range: 34–73), with no significant difference with that of patients who developed post-COVID-19 infection BRVO (*p*=0.82, unpaired *t*-test). The male:female ratio was 6:7. The mean time between COVID-19 vaccination and BRVO symptom onset was 5.1 ± 5.8 days (range: 1–23), which was not significantly different from that of post-COVID-19 vaccination CRVO (Table 2) (*p*=0.10, unpaired *t*-test).

The vaccines used were manufactured by Pfizer, Moderna, and AstraZeneca (Table 5). The mean number of vaccine doses received was 1.4 ± 0.5 (range: 1–2), which was not significantly different from that received by patients with post-COVID-19 vaccination CRVO (Table S2) (*p*=0.14).

The findings were not specific when blood test abnormalities returned to normal levels. The mean logarithmic VA at the initial visit was 0.27 ± 0.34 (range: 0–1), which was not significantly different compared with that of patients with post-COVID-19 infection BRVO (Table 4) (*p*=0.14).

Intravitreal injection of the anti-VEGF antibody is the main treatment. The final logarithmic VA was 0.0.35 ± 0.11(range: −0.079-0.301), with no significant difference compared with that in patients with post-COVID-19 infection BRVO (Table 4) (*p*=0.14).

### 2.5. Findings From Big Data Studies

This systematic review showed that the increased risk of RVOs following COVID-19 is unsurprising, considering their reinforcing effects on each other, particularly in relation to coagulation processes. However, clarifying the relationship between COVID-19 infection or vaccination and RVOs is challenging. Epidemiological investigations make an important contribution to studies on comorbid conditions and the induction of one condition by another.  Table 7 shows big data studies reporting associations between retinal vascular occlusive diseases (RAO and RVO) and COVID-19 infection or vaccination [80–92]. Li et al. analyzed 1,460,634 paired individuals and reported that patients with COVID-19 had a significantly higher risk of BRVO (hazard ratio, 1.27; 95% confidence interval, 1.04–1.52) than those without COVID-19 [80]. The cumulative incidence of BRVO was also significantly higher in patients with COVID-19 (log-rank *p*=0.014) [80]. Modjtahedi et al. reported an RVO incidence of 65 in 432,515 patients (a crude incidence rate of 12.2 per million) at 6 months after COVID-19 diagnosis [81]. This is a clear increase in the rate of RVO compared with that observed in patients not recently infected with COVID-19 [81]. Singh et al. identified 1351 RVO cases globally, with crude reporting rates of 0.36, 0.41, and 0.69 for BNT162b2, mRNA-1273, and Ad26.COV2.S, respectively [82]. Most cases occurred after BNT162b2 (*n* = 606, 74.2%), with a significantly higher risk of RVO onset compared with other vaccines (*p* < 0.0001) [82]. Similarly, Li et al. examined 7,318,437 cases and found that the risk of RVO was significantly elevated during the first 2 weeks after vaccination and persisted for 12 weeks. They also noted an increased risk up to 2 years after the first and second doses of BNT162b2 and mRNA-1273 [83]. Napal et al. reviewed 472 patients with RVO and concluded that the incidence of RVO increased during the first 2 years of the COVID-19 pandemic [84]. Cho et al. analyzed 326,154 patients with RAO and RVO and reported higher RVO incidence during the pandemic, especially in 2021 and 2022 [85]. Conversely, Al-Moujahed reported CRVO diagnosis in 7261 (2.5%) of the 285,759 new patients assessed during the pre-COVID-19 period and 4098 (2.7%) of the 156,427 new patients assessed during the COVID-19 pandemic [86]. In retina clinics, the proportion of newly diagnosed CRVO remained stable during the COVID-19 pandemic [86]. Park et al. reported that the incidence of RVO did not increase after SARS-CoV-2 infection [87, 88]. Dorney et al. analyzed 3,108,829 patients and found no evidence linking mRNA COVID-19 vaccines to new RVO diagnoses [89]. Similarly, Feltgen et al. studied 421 patients with RVO and RAO and reported no association between SARS-CoV-2 vaccination and increased risk of these conditions [90]. Hashimoto et al. also concluded that causality between RVO and COVID-19 vaccination was low [91]. Finally, Rachman et al. were unable to determine differences in the RVO risk across vaccine types due to limited data on dosages and patient histories [92].

### 2.6. Association Between VWF and RVO

VWF has traditionally been described in relation to arterial thrombosis [93]. Michels et al. observed that elevated VWF levels conferred an increased risk of venous thromboembolism and long-term venous complications [94]. Furthermore, Feng et al. reported that increased VWF levels were associated with a prolonged retinal circulation time and reduced retinal blood flow in the early-stage retinopathy of type 1 diabetes [95]. Yamashita et al. investigated the association between VWF and exudative age-related macular degeneration (AMD) and concluded that VWF may play an important role in the pathophysiology of AMD and that aflibercept might improve AMD by reducing plasma levels of VWF [96]. Hirai et al. compared pachychoroid neovasculopathy (PNV) and AMD through analyses focusing on VWF [97]. They found that the residual unusually large VWF multimers may result in platelet thrombosis and hemorrhages in the choriocapillaris of PNV [97]. Thus, VWF may have a deep association with several ocular diseases. However, there are few papers that have investigated the association between VWF and RVO. Boyd et al. reported that plasma homocysteine, methylenetetrahydrofolate reductase C677T and factor II G20210A polymorphisms, factor VIII, and VWF were not identified as new risk factors for CRVO [29]. Hirai et al. found an association between VWF and central choroidal thickness in patients with BRVO [98]. They concluded that the measurement of VWF may be useful for evaluating disease activity and prognosis [98]. These studies [29, 98] show no difference in blood VWF antigen levels in patients with RVO compared with controls. This suggests a difference from RVO that develops in COVID-19 patients with high VWF levels.

### 2.7. COVID-19 Pandemic and RVOs

During the start of the COVID-19 pandemic, the incidence of blood coagulation abnormalities increased greatly. Tang et al. proposed that infected patients with elevated d-dimer levels had a poor prognosis [99]. In fatal cases, the rate of disseminated intravascular coagulation (DIC) was 71.4% [99], indicating that patients with DIC were very unlikely to survive [99]. COVID-19-associated coagulopathy is strongly correlated with vascular endothelial cell damage [24, 25] and increased thrombin production in the veins and arteries. Moreover, d-dimer elevation has been verified as an independent risk factor for thrombosis and death in patients with COVID-19 [100–102]. However, the present study revealed that patients with COVID-19 infrequently have coagulative abnormalities. Wang et al. hypothesized that mild or focal coagulation activation can cause retinal vessel occlusions without a significant change in the patient's d-dimer level [103].

The COVID-19 vaccine approved for clinical use at the end of 2020 is the primary infection control strategy. COVID-19 vaccines have several types, including messenger (m)RNA, DNA, live-attenuated adenovirus vector, inactivated, and recombinant protein vaccines [104]. Solidification fibrinolytic abnormalities after COVID-19 vaccination were reported [105–107]. Vaccine-induced immune thrombocytopenia and thrombosis (VITT) is a new conception and disease after COVID-19 started. Only adenovirus vector vaccines rarely cause VITT [105]. However, an mRNA-based vaccine can cause VITT [106, 107], and carelessness can be very dangerous. Thus, ophthalmologists should closely monitor patients for ocular thrombosis after COVID-19 infection and COVID-19 vaccination.

We showed big data analyses [80–92] in the previous section, and the results are reliable given the large sample sizes. However, the correlation between RVO and COVID-19 infection or vaccination remains unclear.

Herein, we briefly describe two interesting cases we have encountered [108, 109]. The first case was a male patient in his early 50s who had a recurrence of ME due to BRVO 3 days after administering the mRNA-based COVID-19 vaccine (Pfizer-BioNTech). He was administered with an additional intravitreal aflibercept injection [108], and he refused further vaccinations thereafter [108]. No recurrence was reported for 10 months since his initial visit [108]. The second case was a 21-year-old female patient who had been using oral contraceptives for 2 years [109]. She contracted COVID-19 despite receiving two mRNA-based COVID-19 vaccine (Pfizer-BioNTech) doses [109]. Subsequently, she experienced BRVO with ME 40 days after the COVID-19 diagnosis. She was treated with an intravitreal aflibercept injection [109]. ME did not recur for 29 months since the patient's initial visit. BRVO with ME does not usually occur in young women; however, risk factors for venous thromboembolism include oral contraceptive use, COVID-19 vaccination, and COVID-19 infection, all of which could have induced the development of BRVO with ME [109]. These cases illustrate how COVID-19 infection and vaccination can lead to various ocular diseases.

With the global dissemination of COVID-19 vaccines, the pandemic has now (at the end of 2024) been controlled to some degree. However, COVID-19 and its vaccines have several unknown factors, unknown complications, or side effects. Ophthalmologists may identify these complications and side effects in patients after COVID-19 infection or vaccination.

## 3. Conclusions

This systematic review provides a comprehensive summary of RVO cases secondary to either COVID-19 infection or vaccination reported to date. Few cases of RVOs following COVID-19 infection or vaccination have been reported despite their rarity, indicating a major concern. Although clinicians should closely monitor any visual disturbances in patients recently infected with or vaccinated against COVID-19, we can neither support nor reject a possible association between RVOs and COVID-19 infection or vaccination based on existing evidence. The patients in the reviewed studies constitute a heterogeneous sample, and other underlying conditions and/or risk factors for RVOs cannot be ruled out. Therefore, further research with more data is warranted.

## Figures and Tables

**Table 1:** Characteristics of patients with CRVO after a COVID-19 infection.

No.	Authors	Age	Sex	Time between COVID-19 diagnosis and symptom onset (days)	BCVA at the initial visit	Final BCVA
1	Invernizzi et al. [30]	54	F	5	0.3010	0.0000

2	Gaba et al. [31]	40	M	1	0.1739 in the right eye	Not listed
					0.4815 in the left eye	Not listed

3	Riazi-Esfahani et al. [32]	35	M	120	2.6000	Not listed

4	Sheth et al. [33]	52	M	10	1.000	0.1739

5	Walinjkar et al. [34]	17	F	23	0.6021	0.3010

6	Kılıçarslan et al. [35]	50	M	0	2.6000	2.6000

7	Raval et al. [36]	39	M	7	0.8761	0.1739

8	Finn et al. [37]	32	M	30	0.0000	Not listed

9	Lin and Sun [38]	48	M	30	2.6000 (both eyes)	Not listed

10	Yahalomi et al. [39]	33	M	20	0.0969	Not listed

11	Venkatesh et al. [40]	56	F	0	0.4815	0.0000

12	Shroff et al. [41]	41	F	21	1.3010	Not listed

13	Staropoli et al. [42]	15	M	0	2.0000	1.0000

14	Ashkenazy et al. [43]	33	M	42	0.0000	−0.1239
		29	M	84	0.6021	0.0000
		24	F	28	0.4815	0.1739
		36	F	98	0.5436	0.0000
		22	M	63	0.0000	0.0000
		18	F	21	0.0969	0.0000
		50	F	126	0.1739	0.0969
		41	F	105	0.3979	0.1739
		34	M	98	0.0000	0.0000
		30	M	42	2.6000	0.0000
		31	F	7	0.0000	0.0000
		38	F	28	0.0000	0.1739

15	Płatkowska-Adamska et al. [44]	38	M	180	0.6990	0.0000

16	Quigley et al. [45]	42	M	240	0.1739	Not listed

*Note:* F, female; M, male.

Abbreviation: BCVA, best-corrected visual acuity.

**Table 2:** Characteristics of patients with CRVO after a COVID-19 vaccination.

No.	Authors	Age	Sex	Time between vaccination and symptom onset (days)	Vaccine type
1	Sonawane et al. [51]	50	M	4	Oxford-AstraZeneca (ChAdOx1 nCoV-19/AZD1222)
		43	F	3	Oxford-AstraZeneca (ChAdOx1 nCoV-19/AZD1222)

2	Ishiguro et al. [52]	47	M	0 (8 h)	Pfizer/BioNTech (BNT162b2)

3	Lee et al. [53]	34	M	10–12	Pfizer/BioNTech (BNT162b2)

4	Wu et al. [54]	54	M	9	Pfizer/BioNTech (BNT162b2)

5	Romano et al. [55]	54	F	2	Oxford-AstraZeneca (ChAdOx1 nCoV-19/AZD1222)

6	Endo et al. [56]	52	M	15	Pfizer/BioNTech (BNT162b2)

7	Sung et al. [57]	25	F	10	Pfizer/BioNTech (BNT162b2)

8	Dutta Majumder and Prakash [58]	28	M	25	Oxford-AstraZeneca (ChAdOx1 nCoV-19/AZD1222)

9	Shah et al. [59]	27	F	10	Pfizer/BioNTech (BNT162b2)

10	Takacs et al. [60]	35	M	14	mRNA (details unknown)

11	Nangia et al. [61]	13	M	15	Corbevax COVID-19 vaccine

12	Bialasiewicz et al. [62]	50	M	0	Pfizer/BioNTech (BNT162b2)

*Note:* F, female; L, left; M, male; R, right.

**Table 3:** Change in BCVA in patients with CRVO after a COVID-19 vaccination.

No.	Authors	BCVA at the initial visit	Final BCVA
1	Sonawane et al. [51]	1.0000	Not listed
		1.0794	Not listed

2	Ishiguro et al. [52]	1.0000	0.0000

3	Lee et al. [53]	2.6000	0.1759

4	Wu et al. [54]	0.6990	0.3010

5	Romano et al. [55]	1.3010	1.0000

6	Endo et al. [56]	0.0000	0.0000

7	Sung et al. [57]	0.6990	0.1759

8	Dutta Majumder and Prakash [58]	1.4786	0.1759

9	Shah et al. [59]	0.0000	Not listed

10	Takacs et al. [60]	0.3010	0.0000

11	Nangia et al. [61]	0.0969	0.0000

12	Bialasiewicz et al. [62]	0.3010	0.0000

Abbreviation: BCVA, best-corrected visual acuity.

**Table 4:** Characteristics of patients with BRVO after a COVID-19 infection.

No.	Authors	Age	Sex	Time between COVID-19 diagnosis and symptom onset (days)	BCVA at the initial visit	Final BCVA
1	Nourinia et al. [63]	60	F	10	1.0000	Not listed

2	Duff et al. [64]	74	F	90	0.3979	Not listed

3	Karasu and Kesim [65]	48	M	60	0.6990	0.5229
				90	1.0000	0.3979

4	Kapsis et al. [66]	65	M	Not listed	0.7773	0.1759

5	Shiroma et al. [67]	54	F	7	0.0969	0.0000
		36	M	90	0.1759	0.0000
		64	M	7	2.6	1.0000

6	Güven et al. [68]	53	M	Not listed	0.0000	Not listed

*Note:* F, female; M, male.

Abbreviation: BCVA, best-corrected visual acuity.

**Table 5:** Characteristics of patients with BRVO after a COVID-19 vaccination.

No.	Authors	Age	Sex	Time between vaccination and symptom onset (days)	Vaccine type
1	Pur et al. [70]	34	M	2	Pfizer/BioNTech (BNT162b2)

2	Sugihara et al. [71]	38	M	2	Pfizer/BioNTech (BNT162b2)

3	Gironi et al. [72]	50	M	1	Moderna (m-RNA1273)

4	Tanaka et al. [73]	50	F	3	Pfizer/BioNTech (BNT162b2)
		56	F	3	Pfizer/BioNTech (BNT162b2)

5	Karageorgiou et al. [74]	60	M	7	Oxford-AstraZeneca (ChAdOx1 nCoV-19/AZD1222)

6	Lee et al. [75]	41	F	2	mRNA (details unknown)

7	Silva et al. [76]	66	F	16	Oxford-AstraZeneca (ChAdOx1 nCoV-19/AZD1222)

8	Peters et al. [77]	71	M	2	Oxford-AstraZeneca (ChAdOx1 nCoV-19/AZD1222)
		73	F	3	Oxford-AstraZeneca (ChAdOx1 nCoV-19/AZD1222)
		47	F	5	Pfizer/BioNTech (BNT162b2)

9	Choi et al. [78]	66	M	7	Oxford-AstraZeneca (ChAdOx1 nCoV-19/AZD1222)
		69	F	3	Oxford-AstraZeneca (ChAdOx1 nCoV-19/AZD1222)

10	Bolletta et al. [79]	Not listed	Not listed	23	Not listed
		Not listed	Not listed	2	Not listed
		Not listed	Not listed	2	Not listed
		Not listed	Not listed	3	Not listed

*Note:* F, female; L, left; M, male; R, right.

**Table 6:** Change in BCVA in patients with BRVO after a COVID-19 vaccination.

No.	Authors	BCVA at the initial visit	Final BCVA
1	Pur et al. [70]	0.0000	0.0000

2	Sugihara et al. [71]	0.0458	−0.0792

3	Gironi et al. [72]	1.0000	Not listed
		0.1461	Not listed

4	Tanaka et al. [73]	0.0969	0.0000
		0.1871	0.0000

5	Karageorgiou et al. [74]	0.0000	Not listed

6	Lee et al. [75]	0.4776	0.0000

7	Silva et al. [76]	Not listed	Not listed

8	Peters et al. [77]	Not listed	Not listed
		1.0000	Not listed
		0.5006	Not listed

9	Choi et al. [78]	0.0000	Not listed
		0.0000	Not listed

10	Bolletta et al. [79]	0.0000	Not listed
		0.0000	0.0000
		0.6990	0.3010
		0.2041	0.0969
		0.0414	0.0000

Abbreviation: BCVA, best-corrected visual acuity.

**Table 7:** Correlation between RVO and COVID-19 infection or vaccination based on big data studies.

No.	Authors	Number of patients	Conclusions
1	Li et al. [80]	1,460,634 paired patients	Patients with COVID-19 had a significantly higher risk of BRVO

2	Modjtahedi et al. [81]	432,515	Increased incidence of RVO after a COVID-19 infection

3	Singh et al. [82]	2,061,557,270	Significantly higher risk of RVO onset after BNT162b2

4	Li et al. [83]	739,066 paired patients	First and second doses of BNT162b2 and mRNA-1273 were associated with a significantly increased risk of RVO and RAO

5	Napal et al. [84]	472 patients with RVO	Increased incidence of RVO during the first 2 years of the COVID-19 pandemic

6	Cho et al. [85]	304,405 patients with RVO	Incidence of RVO increased during the pandemic, particularly in 2021 and 2022
		10,279 patients with RVO + RAO	

7	Al-Moujahed et al. [86]	442,186	Newly diagnosed CRVO cases remained stable during the COVID-19 pandemic

8	Park et al. [87]	104,090	SARS-CoV-2 infection did not significantly increase RVO or RAO incidence

9	Park et al. [88]	8,418,590	No increase in the hazard ratio of RVO associated with a COVID-19 infection or vaccination

10	Dorney et al. [89]	3,108,829	No evidence of an association between mRNA COVID-19 vaccination and RVO

11	Feltgen et al. [90]	196 patients with RVO	No evidence of an association between SARS-CoV-2 vaccination and increased RVO risk

12	Hashimoto et al. [91]	99,718 paired patients	COVID-19 vaccination was not causally associated with ocular adverse events

13	Rachman et al. [92]	75 patients with RVO	No association could be established between RVO and COVID-19 vaccination

## Data Availability

The datasets used and/or analyzed during this study are available from the corresponding author upon reasonable request.

## Supporting Information

Table S1 shows the summary of patients with CRVO after a COVID-19 infection.

Table S2 shows the characteristics of patients with CRVO after a COVID-19 vaccination.

Table S3 shows the summary of patients with BRVO after a COVID-19 infection.

Table S4 shows the characteristics of patients with BRVO after a COVID-19 vaccination.
