# Supplementary material for: Relationship Between COVID-19 and Retinal Vein Occlusions
Source: J Ophthalmol. 2025 Sep 29;2025:6507997. doi: 10.1155/joph/6507997 (PMC12500366; doi:10.1155/joph/6507997)
Supplement: Supporting Information — Additional supporting information can be found online in the Supporting Information section. [file 6507997.f1.docx]

**Table S1. Summary of patients with CRVO after COVID-19 infection**

| **No.** | **Authors** | **Laterality** | **Abnormal blood test findings** | **Treatment** |
| --- | --- | --- | --- | --- |
| 1 | Invernizzi et al. [30] | R | CRP (31.1 mg/L) | Oral prednisolone (60 mg/day) |
|  |  |  | ESR (78 mm/h) |  |
|  |  |  | Lactate dehydrogenase (269 U/L) |  |
|  |  |  | PT (13.8 s) |  |
|  |  |  | aPTT (36.6 s) |  |
|  |  |  | Fibrinogen (6.82 g/L) |  |
|  |  |  | d-Dimer (426 μg/L) |  |
| 2 | Gaba et al. [31] | R, L | Ferritin (1518 μg/L) | Rivaroxaban (15 mg twice daily) |
|  |  |  | LDH (402 U/L) |  |
|  |  |  | d-Dimer (>20 μg/L) |  |
|  |  |  | CRP (68 mg/L) |  |
|  |  |  | Interleukin-6 (87.1 pg/mL) |  |
| 3 | Riazi-Esfahani et al. [32] | L | CRP borderline | Three doses of IV anti-VEGF |
|  |  |  | Homocysteine borderline |  |
| 4 | Sheth et al. [33] | L | Unremarkable | Oral methylprednisolone (40 mg/day) |
|  |  |  |  | IV ranibizumab BS |
| 5 | Walinjkar et al. [34] | R | Not listed | IVR |
| 6 | Kılıçarslan et al. [35] | R | PPT (20.3 s) | IVA and systemic steroid therapy |
|  |  |  | LDH (222 U/L) |  |
| 7 | Raval et al. [36] | R | Unremarkable | IVB |
| 8 | Finn et al. [37] | R | Not listed | Not listed |
| 9 | Lin et al. [38] | R, L | d-Dimer (1050 μg/L) | IV anti-VEGF |
|  |  |  | CRP (86.89 mg/L) |  |
| 10 | Yahalomi et al. [39] | L | Unremarkable | Not listed |
| 11 | Venkatesh et al. [40] | L | d-Dimer (707 μg/L) | Oral aspirin |
|  |  |  | ESR (52 mm) |  |
| 12 | Shroff et al. [41] | R | d-Dimer (0.9 μg/L) | IV anti-VEGF |
| 13 | Staropoli et al. [42] | L | None | Doxycycline (100 mg twice daily) |
|  |  |  |  | Prednisolone eye drops |
|  |  |  |  | IVB |
| 14 | Ashkenazy et al. [43] |  | Not listed |  |
|  |  |  | Not listed | IVB |
|  |  |  | Not listed |  |
|  |  |  | Not listed | IVB |
|  |  |  | Not listed |  |
|  |  |  | Not listed |  |
|  |  |  | Not listed | Plavix |
|  |  |  | Not listed |  |
|  |  |  | Not listed |  |
|  |  |  | Not listed | Oral prednisolone |
|  |  |  |  | Oral aspirin |
|  |  |  |  | IVB |
|  |  |  | Not listed |  |
|  |  |  | Not listed | IVB |
| 15 | Płatkowska-Adamska et al. [44] | R | Cholesterol (243 mg/dL) | IVR |
|  |  |  | d-Dimer (543 ug/L) |  |
| 16 | Quigley et al. [45] | R | WBC count (3.5 × 10^9^/L) | IVB |
|  |  |  | Total cholesterol (5.4 mmol/L) |  |
|  |  |  | Neutrophil count (1.4 × 109/L) |  |

aPTT, activated partial thromboplastin time; BS, biosimilar; CRP, C-reactive protein; ESR, erythrocyte sedimentation rate; IV, intravitreal; IVA, intravitreal aflibercept; IVB, intravitreal bevacizumab; IVR, intravitreal ranibizumab; L, left; LDH, lactate dehydrogenase; PT, prothrombin time; PPT, partial prothrombin time; R, right; VEGF, vascular endothelial growth factor; WBC, white blood cell.

Note: IV anti-VEGF indicates cases in which the specific anti-VEGF drug administered was not reported.

Table S2. Characteristics of patients with CRVO after COVID-19 vaccination

| **No.** | **Authors** | **Laterality** | **Vaccine doses** | **Abnormal blood test findings** | **Treatment** |
| --- | --- | --- | --- | --- | --- |
| 1 | Sonawane et al. [51] | R | Second | HbA1c (13.2%) | IV anti-VEGF |
|  |  | R | Second | Cre (1.9 mg/dL) | None |
|  |  |  |  | ESR (49mm/h) |  |
|  |  |  |  | CRP (14.6) |  |
|  |  |  |  | RF (11) |  |
|  |  |  |  | d-Dimer (6,077.4 ng/mL) |  |
| 2 | Ishiguro et al. [52] | R | First | Triglyceride (256 mg/dL) | IVA |
|  |  |  |  | aPTT (30.6 s) |  |
|  |  |  |  | CRP (177%) |  |
| 3 | Lee et al. [53] | L | Second | Total cholesterol (227 mg/dL) | Oral methylprednisolone |
|  |  |  |  | LDL (159) | IV methylprednisolone |
|  |  |  |  | ESR (26) |  |
| 4 | Wu et al. [54] | L | Second | Not listed | IVB |
| 5 | Romano et al. [55] | R | Second | Normal | IV dexamethasone  implant |
| 6 | Endo et al. [56] | L | First | Normal | IV dexamethasone |
|  |  |  |  |  | IVB, oral apixaban |
| 7 | Sung et al. [57] | L | Third | HbA1c (9.2%) | IVR, IVA |
| 8 | Dutta Majumder et al. [58] | R | Third | Normal | IVB, oral apixaban |
|  |  |  |  |  | Oral corticosteroid |
| 9 | Shah et al. [59] | L | First | Not listed | IVR |
|  |  |  |  |  | Acetazolamide |
|  |  |  |  |  | intravenous iron infusions |
| 10 | Takacs et al. [60] | R | First | Serum PT (9.1 s) | IVA |
|  |  |  |  | Anti-prothrombin (123%) | Oral ASA protect |
|  |  |  |  | Serum homocysteine (16.4 μmol/L) |  |
| 11 | Nangia et al. [61] | L | First | Normal | Pulse corticosteroid |
| 12 | Bialasiewicz et al. [62] | L | Second | Normal | Aspirin |
|  |  |  |  |  | IVA |

aPTT, activated partial thromboplastin time; ASA; acetylsalicylic acid (aspirin); Cre, creatinine; CRP, C-reactive protein; ESR, erythrocyte sedimentation rate; HbA1c, hemoglobin A1c; IV, intravitreal; IVA, intravitreal aflibercept; IVB, intravitreal bevacizumab; IVR, intravitreal ranibizumab; L, left; LDL, low-density lipoprotein; PT, prothrombin time; R, right; RF, rheumatoid factor; VEGF, vascular endothelial growth factor.

Note: IV anti-VEGF indicates cases in which the specific anti-VEGF drug administered was not reported.

**Table S3. Summary of patients with BRVO after COVID-19 infection**

| **No.** | **Authors** | **Laterality** | **Abnormal blood test findings** | **Treatment** |
| --- | --- | --- | --- | --- |
| 1 | Nourinia et al. [63] | L | Slightly prolonged PT and PTT | IVB |
|  |  |  | High ESR level (up to 76) | Intravitreal dexamethasone implant |
|  |  |  | High CRP level  (up to 129 mg/L) |  |
|  |  |  | High d-Dimer level  (up to 0.76 g/mL) |  |
|  |  |  | High ferritin level  (up to 430 ng/mL) |  |
|  |  |  | Elevated WBC (up to 17,700) |  |
| 2 | Duff et al. [64] | L | Not listed | Heparin |
| 3 | Karasu et al. [65] | L | Not listed | Pantoprazole |
|  |  | R | Not listed | Favipiravir |
| 4 | Kapsis et al. [66] | L | Normal | IVA |
| 5 | Shiroma et al. [67] | Not listed | Not listed | Ketorolacid |
|  |  | Not listed | Not listed | IV anti-VEGF |
|  |  | Not listed | Not listed | IV anti-VEGF |
| 6 | Güven et al. [68] | L | ESR (74 mm/h) | Not listed |
|  |  |  | CRP (29.8 mg/L) |  |
|  |  |  | d-Dimer (404 μg/L) |  |

aPTT, activated partial thromboplastin time; CRP, C-reactive protein; ESR, erythrocyte sedimentation rate; IV, intravitreal; IVA, intravitreal aflibercept; IVB, intravitreal bevacizumab; L, left; PT, prothrombin time; PPT, partial prothrombin time; R, right; VEGF, vascular endothelial growth factor; WBC, white blood cell.

Note: IV anti-VEGF indicates cases in which the specific anti-VEGF drug administered was not reported.

Table S4. Characteristics of patients with BRVO after COVID-19 vaccination

| **No.** | **Authors** | **Laterality** | **Amount of vaccine doses** | **Abnormal blood test findings** | **Treatment** |
| --- | --- | --- | --- | --- | --- |
| 1 | Pur et al. [70] | R | First | Normal | Observation |
| 2 | Sugihara et al. [71] | L | Second | Normal | Second dose of IVA |
| 3 | Gironi et al. [72] | R | Booster dose | Mild alteration in the liver function | IVR PC |
|  |  | L |  |  | IVR PC |
| 4 | Tanaka et al. [73] | R | First | Not listed | Three doses of IVR |
|  |  | R | First | Not listed | Three doses of IVR |
| 5 | Karageorgiou et al. [74] | R | Not listed | Normal | Not listed |
| 6 | Lee et al. [75] | R | Second | ESR 46 mm/h | Three doses of IVR |
| 7 | Silva et al. [76] | R | Not listed | CRP 1.0 mg/dL | Not listed |
| 8 | Peters et al. [77] | Not listed | First | Not listed | Not listed |
|  |  | Not listed | First | Not listed | IVB |
|  |  | Not listed | First | Not listed | IVA |
| 9 | Choi et al. [78] | L | Second | Not listed | IVB |
|  |  | L | First | Not listed | Observation |
| 10 | Bolletta et al. [79] | L | Second | Not listed | Oral aspirin |
|  |  | L | First | Not listed | IV anti-VEGF |
|  |  | L | Second | Not listed | IV anti-VEGF |
|  |  | L | Second | Not listed | IV anti-VEGF |

CRP, C-reactive protein; ESR, erythrocyte sedimentation rate**;** IV, intravitreal; IVA, intravitreal aflibercept; IVB, intravitreal bevacizumab; IVR, intravitreal ranibizumab; L, left; PC, photocoagulation; R, right; VEGF, vascular endothelial growth factor.

Note: IV anti-VEGF indicates cases in which the specific anti-VEGF drug administered.
